# Supplementary material for: The Related Mechanisms Predicted through Network-Based Pharmacological Analysis and the Anti-Inflammatory Effects of Fraxinus rhynchophylla Hance Bark on Contact Dermatitis in Mice
Source: Int J Mol Sci. 2023 Mar 23;24(7):6091. doi: 10.3390/ijms24076091 (PMC10094049; doi:10.3390/ijms24076091)
Supplement: Supplementary file 1 [file ijms-24-06091-s001.zip › ijms-2263352-supplementary/Supplementary Materials S1.pdf]

Supplementary data

Table S1. Total compounds of *F. rhynchophylla*

No.	Compound name	PubChem CID	Molecular weight	OB (%)	DL
1	(2S,3R,4S,5S,6R)-2-[4-[(3R,3aS,6S,6aR)-3a-hydroxy-6-(4-hydroxy-3-methoxyphenyl)-3,4,6,6a-tetrahydro-1H-furo[3,4-c]furan-3-yl]-2-methoxyphenoxy]-6-(hydroxymethyl)oxane-3,4,5-triol	14756314	536.5	28.54	0.86
2	2,6-Dimethoxy-1,4-benzoquinone (DMBQ)	68262	168.15	12.00	0.04
3	8-Hydroxypinoresinol	3010930	374.40	92.43	0.55
4	Beta-sitosterol	222284	414.70	36.91	0.75
5	Caffeic acid	689043	180.16	25.76	0.05
6	Esculetin	5281416	178.14	22.97	0.07
7	Esculin	5281417	340.28	20.43	0.36
8	Fraxetin	5273569	208.17	23.04	0.09
9	Fraxin	5273568	370.31	36.76	0.42
10	Melissic acid	10471	452.80	13.22	0.49
11	Nonox D	8679	219.28	39.82	0.13
12	Osmanthuside H	192437	432.40	8.89	0.57
13	Scopoletol	5280460	192.17	27.77	0.08
14	Sinapaldehyde	5280802	208.21	63.16	0.07
15	Sinapaldehyde glucoside	25791064	370.40	20.91	0.33
16	Sinapyl alcohol	5280507	210.23	10.23	0.07
17	Sitogluside	5742590	576.80	20.63	0.62
18	Syrigin	5316860	372.40	14.64	0.32
19	Syringaldehyde	8655	182.17	67.06	0.05
20	Tyrosol	10393	138.16	33.81	0.02
21	Ursolic acid	64945	456.70	16.77	0.75
