# Supplementary material for: The Related Mechanisms Predicted through Network-Based Pharmacological Analysis and the Anti-Inflammatory Effects of Fraxinus rhynchophylla Hance Bark on Contact Dermatitis in Mice
Source: Int J Mol Sci. 2023 Mar 23;24(7):6091. doi: 10.3390/ijms24076091 (PMC10094049; doi:10.3390/ijms24076091)
Supplement: Supplementary file 1 [file ijms-24-06091-s001.zip › ijms-2263352-supplementary/Supplementary Materials S2.pdf]

Supplementary data

The effects of *Fraxinus rhynchophylla* Hance bark ethanol extract on ICAM-1 expression in HaCaT keratinocytes

Materials and Methods

Preparation of F. rhynchophylla bark extract

F. rhynchophylla bark was purchased from Kwangmyungdang drug company (Ulsan, Korea). The dried bark of *F. rhynchophylla* (200 g) was immersed in 1L of 70% ethanol, sonicated for 15 min, and then after macerated for 24 h. The next day, 70% ethanol was replaced and the same extraction method was repeated. The extract was filtered through Whatman filter paper (No. 20, Tokyo Roshi Kaisha, Tokyo, Japan) and evaporated under reduced pressure using a vacuum evaporator (Eyela Co., Ltd., Tokyo, Japan). The concentrated extract was lyophilized using a freeze dryer (Labconco, Kansas City, MO, USA). Finally, 6.5 g of lyophilized powder was obtained (EEFR, yield 3.25%).

Cell culture

HaCaT cells were cultured at 37℃ and 5% CO_2 in Dulbecco's modified Eagle's Medium (DMEM) containing L-glutamine (HyClone, UT, USA) supplemented with 10% fetal bovine serum (FBS; HyClone, UT, USA) and 1% penicillin and streptomycin (Gibco, CA, USA).

Cytotoxicity

Cell viability was evaluated using a 3-[4,5-dimethylthiazol-2-yl]-2, 5-diphenyltetrazolium bromied (MTT) assay. Briefly, HaCaT cells ($1x10^4$ cells/well) were seeded into each well of a

24 well cell culture plate (SPL, Seoul, Korea) and incubated at 37°C with 5% CO_2 for 24 h. Cells were then incubated with different concentrations of EEFR for 24 h. After incubation, MTT solution (0.5 mg/ml) was added to each well and incubated for 2 h at 37°C. Dimethyl sulfoxide (DMSO; Bio Basic Inc, CANADA) was then added to solubilize the purple formazan crystals. Finally, the absorbance was measured at 570 nm using a spectrophotometer (Tecan, Infinite® M200, Switzerland).

TNF-α stimulation in HaCaT cell

HaCaT cells were seeded in 60 mm cell culture plate (SPL, Seoul, Korea) (3 x 10^5 cells/plate). After overnight incubation, cells were then treated with 200 μg/ml of EEFR for 24 h. Following incubation, cells were stimulated using 10 ng/ml of TNF-α (Bio-techne, Minneapolis, MN, USA) for 15 min, then harvested and analyzed by western blotting.

Reverse transcription PCR

Prior to stimulate HaCaT cells with TNF-α, cells were pre-treated with 200 μg/ml of EEFR for 1 h, then stimulated with 10 ng/ml of TNF-α for 4 h. Following incubation, total RNA was extracted using TRlsureTM (Bioline, Luckenwalde, Germany) according to the manufacturer's instructions. Briefly, 2 μg of total RNA were used to prepare cDNA by using M-MLV cDNA synthesis kit (Enzynomics, Daejeon, Korea). PCR was then performed with under the following conditions; initial denaturation at 95°C for 5 min, followed by 26 cycles of 95°C for 30 sec, 55°C for 30 sec, 72°C for 40 sec and then final extension at 72°C for 7 min. Aliquots of the PCR products were subsequently electrophoresed in 1.5% agarose gels containing gel RED and visualized under UV using a gel documentation system (BioRad Gel Doc1000 and Multi-

Analyst® version 1.1; BioRad, Richmond, CA, USA). Sequences of primers are summarized in table 2.

Table. S2. Oligonucleotide primers used for the PCR analysis

Target gene	Oligonucleotide sequences (5' to 3' direction)	Product size
ICAM-1	CATCGGGGAATCAGTGACTG<br>TGGGCCTGTTGTAGTCTGGG	234 bp
GAPDH	AGGTCGGAGTCAACGGATTT<br>ATCTCGCTCCTGGAAGATGG	230 bp

Evaluation of ICAM-1 expression in HaCaT cells by Flow cytometry (FACS)

HaCaT cells were treated with 50, 100, and 200 μg/ml of EEFR for 1 h, then stimulated using 10 ng/ml of TNF-α for 23 h. After stimulation, cells were washed with PBS and harvested from 0.05% trypsin-EDTA (Gibco, USA). Harvested cells were subsequently suspend and blocked in 0.5% bovine serum albumin (BSA; Sigma aldrich, St.,Louis, MO) solution buffer for 30 min at room temperature. The supernatant was then removed from the centrifuge (4℃, 400 g, 3 min) and incubated with primary antibodies of rabbit polyclonal ICAM-1 (1:100) (Santa Cruze, CA, USA) for 1 h at room temperature. After washing, cells were incubated with Alexa Fluor 488 conjugated anti-rabbit IgG (Cell signaling, USA) for 30 min, and then fixed in 4% paraformaldehyde solution (LPS solution, Daejeon, Korea). Samples were then evaluated by flow cytometry (BD FACS Canto II. San Diego, CA, USA).

Statistical analysis

All data were analysed using one-way ANOVA followed by Dunnett's multiple comparison test. Prime 5 for Windows version 5.01 (GraphPad Software Inc., La Jolla, CA, USA) was employed for all analysis. All data were presented as the means ± standard deviation and a $P < 0.05$ was considered significant.

Results

EEFR did not affect cell viability in HaCaT cells at concentrations up to 200 μg/ml

When HaCaT cells were exposed to 0, 50, 100, 200 and 400 μg/ml of EEFR for 24 h, EEFR did not show a significant cytotoxicity against HaCaT cells at concentrations up to 200 μg/ml (Fig. 1).

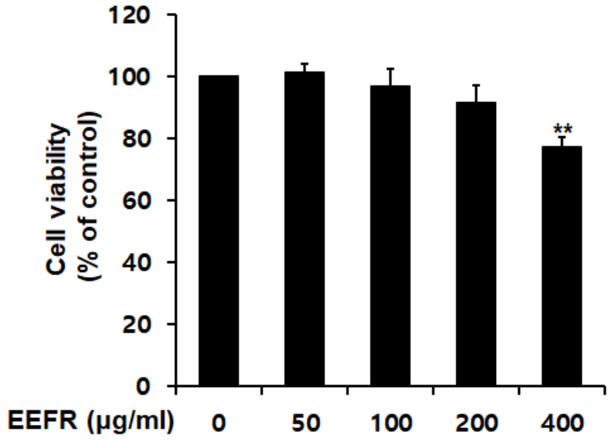

Figure S1. Effects of EEFR on the cell viabilities of HaCaT cells Cell viability was measured by MTT assay. Cells were treated with EEFR at concentrations of 0, 50, 100, 200 and 400 μg/ml for 24 h. Values were represented as mean ± standard deviation. $^{**}P < 0.01$ vs. CTL.

EEFR suppressed ICAM-1 RNA expression

TNF-α (10 ng/ml) treatment induced overexpression of ICAM-1 RNA in HaCaT cells. Treatment with EEFR (200 µg/ml) suppressed the TNF-α induced ICAM-1 RNA expression relative to the non-stimulated group (Fig. 3).

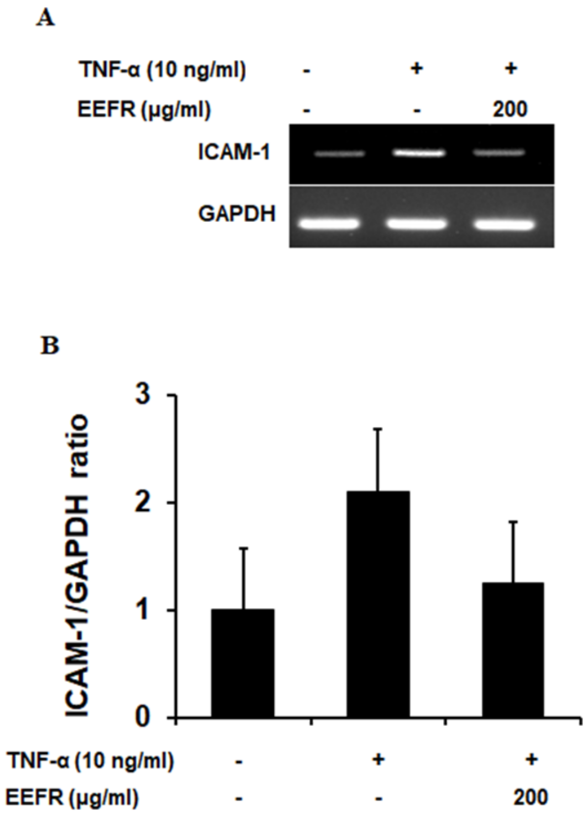

Figure S2. The effects of EEFR on the ICAM-1 RNA expression in HaCaT cells HaCaT cells were treated with EEFR (200 µg/ml) for 1 h, then stimulated with TNF-α (10 ng/ml) for 4 h. The ICAM-1 RNA expression was measured using reverse transcription PCR. GAPDH was used as a loading control. Values are indicated as mean ± standard deviation.

EEFR inhibited ICAM-1 expression

Treatment with TNF-α (10 ng/ml) markedly induced the surface expression of ICAM-1 protein. These increased expression was effectively suppressed by EEFR treatment (Fig. 4).

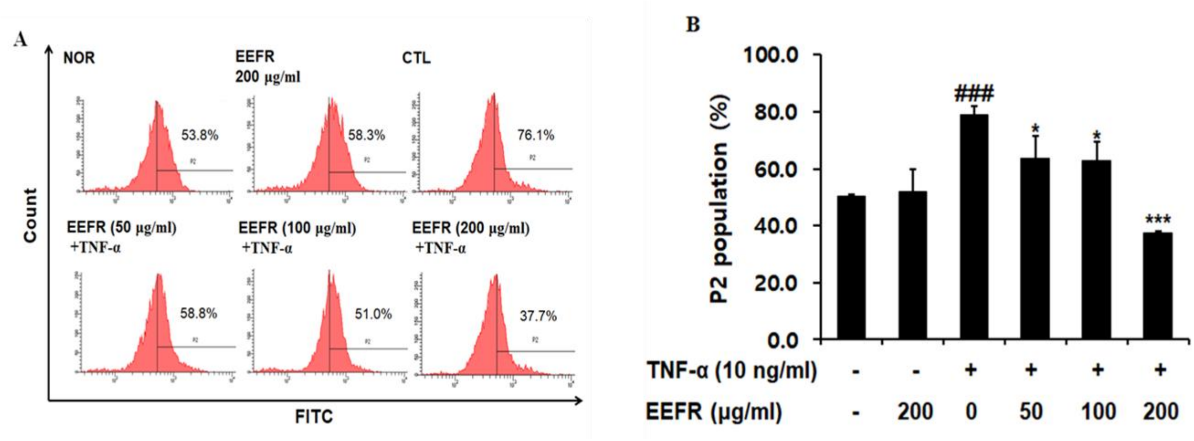

Figure S3. The effects of EEFR on ICAM-1 expression in HaCaT cells. The suppressive effects of EEFR on ICAM-1 overexpression by TNF- α were evaluated using flow cytometric analysis. Data are presented as histograms (A) and bar graphs that evaluated the percentage of the P2 population (B). Values were represented as mean ± standard deviation. $^{\#\#\#}$P < 0.001 vs. NOR; *P < 0.05 and ***P < 0.001 vs. CTL.

Conclusions

These results indicate that EEFR can suppress ICAM-1 expression induced by TNF- α stimulation.
