# Supplementary material for: The Related Mechanisms Predicted through Network-Based Pharmacological Analysis and the Anti-Inflammatory Effects of Fraxinus rhynchophylla Hance Bark on Contact Dermatitis in Mice
Source: Int J Mol Sci. 2023 Mar 23;24(7):6091. doi: 10.3390/ijms24076091 (PMC10094049; doi:10.3390/ijms24076091)
Supplement: Supplementary file 1 [file ijms-24-06091-s001.zip › ijms-2263352-supplementary/Supplementary Materials S3.pdf]

Supplementary data

Esculin did not affect skin lesions in mice with Contact dermatitis

Introduction

Esculin, a coumarin glucoside, is one of the major bioactive constituents isolated from bark of *Fraxinus rhynchophylla* which is a commonly used herbal medicine in Asian countries. Esculetin is an aglycone metabolite of esculin and is one of the simplest coumarins, with two hydroxyl groups at carbons 6 and 7. These two bioactive components have strong anti-oxidant, anti-inflammatory, anti-cancer and photo-protective activities (Rehman et al., 2015). Recently, esculetin has been reported to attenuate atopic skin inflammation induced by house dust mite and 2,4-dinitrochlorobenzene (Jeong et al., 2018). In addition, Chen et al have reported that esculetin can ameliorate psoriasis like skin disease in mice (Chen et al., 2018).

We investigated whether esculin can attenuate skin lesions of contact dermatitis (CD) induced by 1-fluoro-2,4-dinitrobenzene (DNFB) in mice. To accomplish this, the effects of esculin on surface symptoms of CD, changes in skin color, skin thickness were evaluated.

Materials and Methods

Preparation of esulin

Esculin (purity ≥ 98.0%) was purchased from Merck Millipore (KGaA, Darmstadt, Germany). Esculin was dissolved in ethanol then, treated at a concentration of 600 μg/day for 6 consecutive days.

Animals

Male 6-week-old Balb/c mice were purchased from Samtaco (Osan, South Korea). Mice were housed under specific pathogen-free conditions with a 12 hours light/dark cycle and free access to standard rodent food and water. All animal experiments were approved by animal care and use committee and conducted according to institutional guidelines (PNU-2015-0979).

Induction of CD and experimental design

CD was induced by DNFB in Balb/c mice as previously described (Yang et al., 2019). Briefly, mice were sensitized with 30 μl of DNFB (0.2%, v/v) in acetone: olive oil (AOO, 4:1) onto both ears for three consecutive days. After four days of sensitization, the dorsum of all mice were shaved. On day 8, each mouse was challenged with 60 μl of DNFB (0.1% v/v) in AOO onto the shaved dorsum every two days (four times applications in total).

For the treatment with esculin and dexamethasone (DEX), mice were randomly divided into four groups; normal (NOR) group, non-treated normal mice (n=6); control (CTL) group, non-treated CD mice (n=8); esculin group, 600 μg/day of esculin treated CD mice (n=8); DEX group, 150 μg/day of DEX treated CD (n=8). The experimental schedule is summarized in Fig. 1.

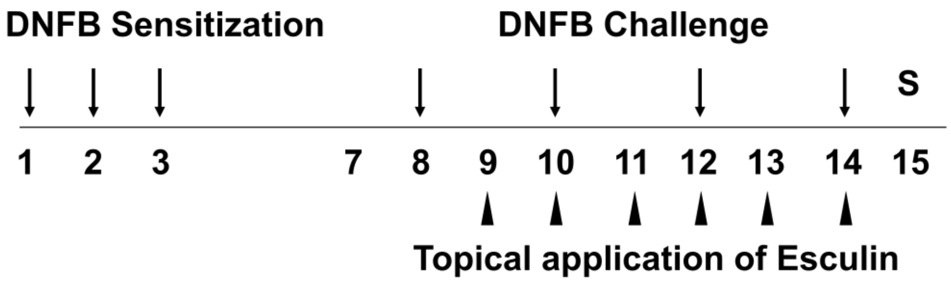

Figure S1. Experimental schedule. DNFB was applied onto the surface of each ear for sensitization on days 1, 2 and 3. Challenging was conducted using 0.1% of DNFB on days 8, 10, 12 and 14. Esculin and dexamethasone were topically applied for 6 consecutive days. All animals were sacrificed on day 15. S, sacrifice.

Observation of skin surface

Skin lesions of mice were observed on day 15 using digital camera (Olympus, Tokyo, Japan). The main subjects of observation were skin lesions such as scaling, peeling, erythema, and roughness of each site with individual symptoms.

Measurement of skin thicknesses

The skin tissue was resected pieces (5 mm in diameter). Thicknesses was then measured using vernier calipers (Mitutoyo, Kanagawa, Japan).

Measurement of erythema and melanin index

The erythema and melanin index were measured using dermo-spectrophotometer (Cortex Technology, Hadsund, Denmark). The erythema and melanin index of each mouse was

calculated using measurements obtained from three different points on the skin surface of each mouse.

Measurement of body and spleen weights

Body weights of each mouse were measured on day 1 and day 15 using electronic scale (CAS, Gyeonggi, Korea) respectively. Changes in body weights were expressed as percentages of weight on day 1. Spleen weights were measured on day 15 using microbalance (Sartorius, Gyeonggi, Korea). The effects of EEFR on spleen weights are presented as the spleen body weight ratio.

Statistical analysis

All data were analysed using one-way ANOVA followed by Dunnett's multiple comparison test. Prime 5 for Windows version 5.01 (GraphPad Software Inc., La Jolla, CA, USA) was employed for all analysis. All data were presented as the means ± standard deviation and a $P < 0.05$ was considered significant.

Result

Esculin did not affect surface skin lesions in CD mice

Repeated stimulation with DNFB induced skin lesions of CD such as scaling, excoriation, erythema and skin roughness. These symptoms were not affected by esculin and DEX (Fig. 2).

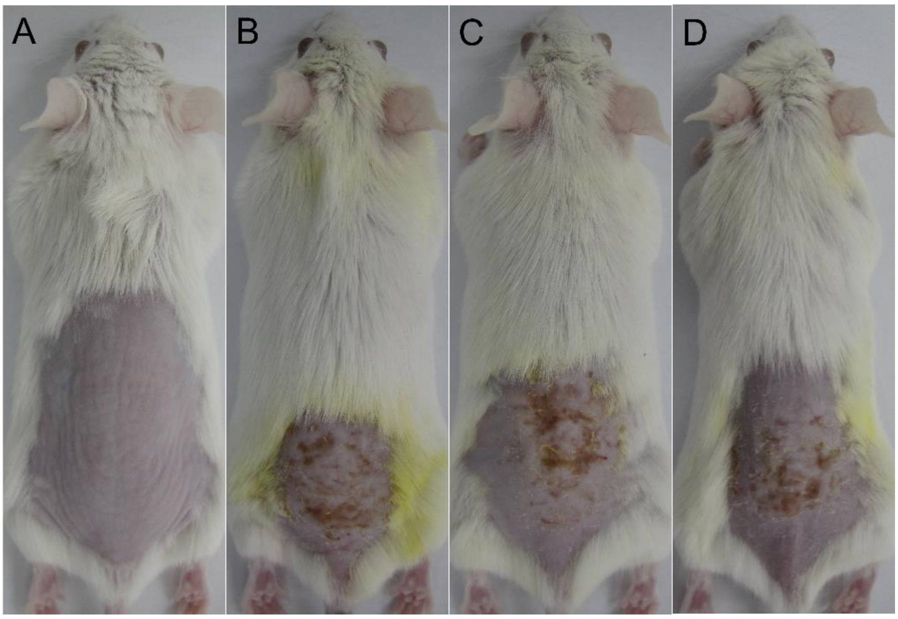

Figure S2. The effects of esculin on skin lesions in CD mice. Skin lesions in the NOR (A), CTL (B), 600 μg/day of esculin (C) and 150 μg/day of DEX groups (D) were observed using a digital camera.

Esculin did not affect enlargement of skin thickness in CD mice

Topical application of DNFB induced increases in skin thickness which is a feature of CD. The skin thickness in CTL group was increased significantly compared to that in the NOR group. Esculin did not affect skin enlargement in CD mice. DEX treatment prevented enlargement of skin thickness significantly (Fig. 3).

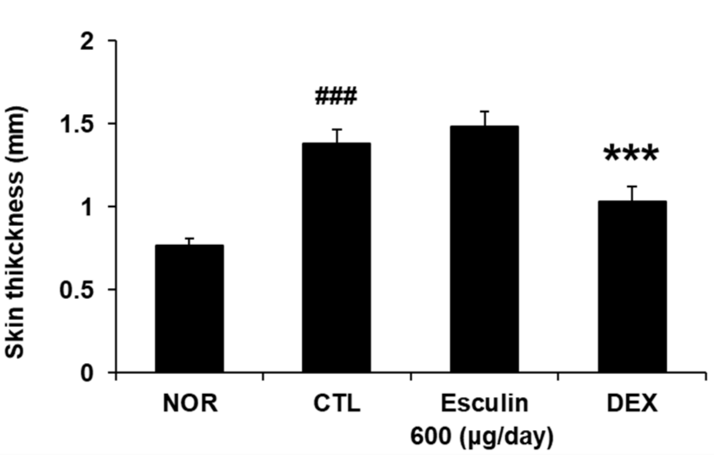

Figure S3. The effects of esculin on skin thickness in CD mice. Skin thicknesses were measured using a vernier calipers. NOR, non-treated normal mice; CTL, non-treated CD mice; Esculin, 600 μg/day of esculin treated CD mice; DEX, 150 μg/day of dexamethasone treated CD mice. Values were represented as mean ± standard deviation. $^{###}P < 0.001$ vs. NOR and $^{**}P < 0.01$ vs. CTL.

EEFR did not affect skin color in CD mice

DNFB stimulation elevated the erythema index significantly in the CTL group. Topical application of 600 μg/day esculin did not affect erythema index. The mice in all experimental groups showed similar levels of melanin index (Fig. 4).

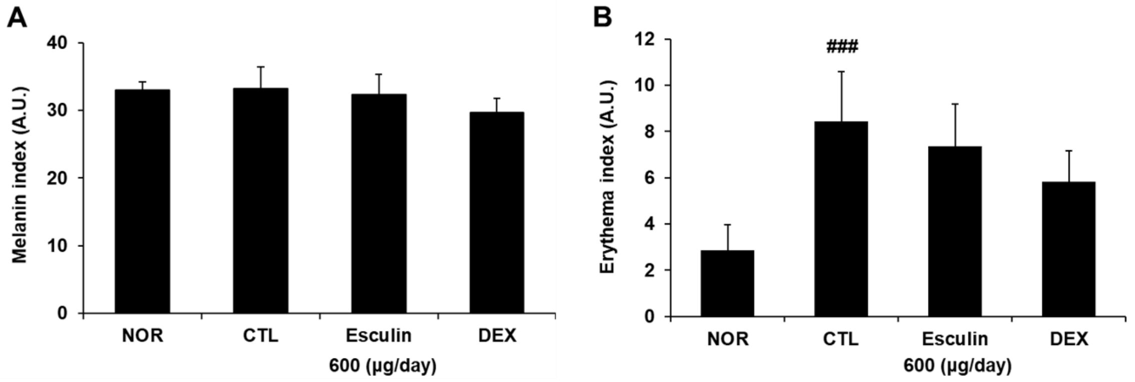

Figure S4. Effects of esculin on erythema and melanin index. Erythema index (A) and melanin index (B) were measured using dermo-spectrophotometer. Abbreviations are same as Fig. 3. Values were represented as mean ± standard deviation. $^{###}P < 0.001$ vs. NOR.

Esculin did not affect spleen enlargement.

The effect of esculin on spleen enlargement was estimated by determining spleen/body weight ratio. The inflammatory response induced by DNFB stimulation caused spleen

enlargement. The spleen/body weight ratios in the esculin treated groups were similar to that of the CTL group. DEX treatment lowered spleen body weight ratio significantly. Even spleen body weight ratio in the DEX group was lower than that in the normal group. (Fig. 5).

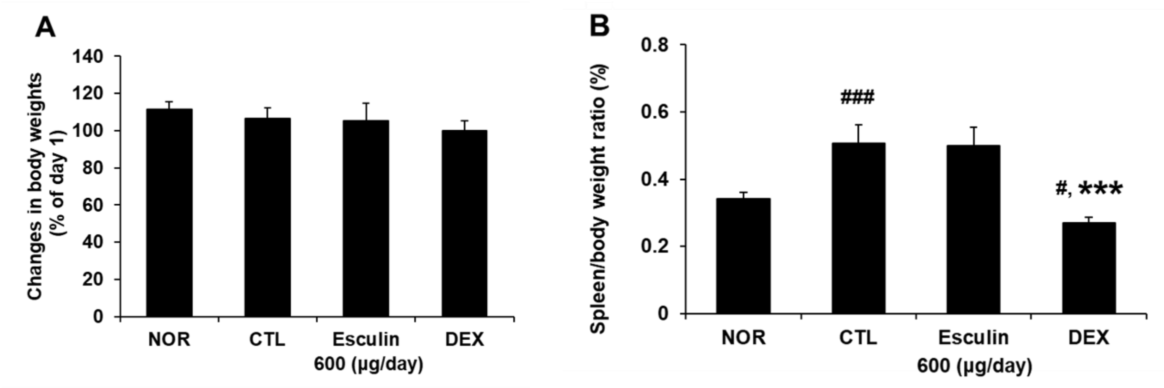

Figure S5. Effects of Esculin on spleen/body weight ratio in CD mice. Body and spleen weight were measured on day 15 and the spleen/body weight ratio was calculated. (A), changes in body weights; (B), spleen/body weight ratio. Abbreviations are same as Fig. 3. $^{\#}P < 0.05$ and $^{\#\#\#}P < 0.001$ vs. NOR; $^{***}P < 0.001$ vs. CTL.

Conclusions

This study showed that esculine did not affect skin lesions, thickness and color in mice with CD differently than esculetin. These results imply that the therapeutic efficacy of *F. rhynchophylla* is closely related to active ingredients such as esculetin rather than esculin.

References

27. Jeong, N.H.; Yang, E.J.; Jin, M.; Lee, J.Y.; Choi, Y.A.; Park, P.H.; Lee, S.R.; Kim, S.U.; Shin, T.Y.; Kwon, T.K.; et al. Esculetin from Fraxinus rhynchophylla attenuates atopic skin inflammation by inhibiting the expression of inflammatory cytokines. Int. Immunopharmacol. 2018, 59, 209–216. https://doi.org/10.1016/j.intimp.2018.04.005.

28. Chen, Y.; Zhang, Q.; Liu, H.; Lu, C.; Liang, C.L.; Qiu, F.; Han, L.; Dai, Z. Esculetin Ameliorates Psoriasis-Like Skin Disease in Mice by Inducing CD4+Foxp3+ Regulatory T Cells. Front. Immunol. 2018, 9, 2092. https://doi.org/10.3389/fimmu.2018.02092.

30. Yang, B.; Kim, S.; Kim, J.H.; Lim, C.; Kim, H.; Cho, S. Gentiana scabra Bunge roots alleviates skin lesions of contact dermatitis in mice. J. Ethnopharmacol. 2019, 233, 141–147. https://doi.org/10.1016/j.jep.2018.12.046.
