# Supplementary material for: The Related Mechanisms Predicted through Network-Based Pharmacological Analysis and the Anti-Inflammatory Effects of Fraxinus rhynchophylla Hance Bark on Contact Dermatitis in Mice
Source: Int J Mol Sci. 2023 Mar 23;24(7):6091. doi: 10.3390/ijms24076091 (PMC10094049; doi:10.3390/ijms24076091)
Supplement: Supplementary file 1 [file ijms-24-06091-s001.zip › ijms-2263352-supplementary/Supplementary Materials S4.pdf]

Supplementary data

Measurement of body and spleen weights

Body weights of each mouse were measured on day 1 and day 15 using electronic scale (CAS, Gyeonggi, Korea) respectively. Changes in body weights were expressed as percentages of weight on day 1. Spleen weights were measured on day 15 using microbalance (Sartorius, Gyeonggi, Korea). The effects of EEFR on spleen weights are presented as the spleen body weight ratio.

EEFR did not affect spleen enlargement.

The effect of EEFR on spleen enlargement was estimated by determining spleen/body weight ratio. The inflammatory response induced by DNFB stimulation caused spleen enlargement. The spleen/body weight ratios in the EEFR treated groups were similar to that of the CTL group. DEX treatment lowered spleen body weight ratio significantly (Fig. 7).

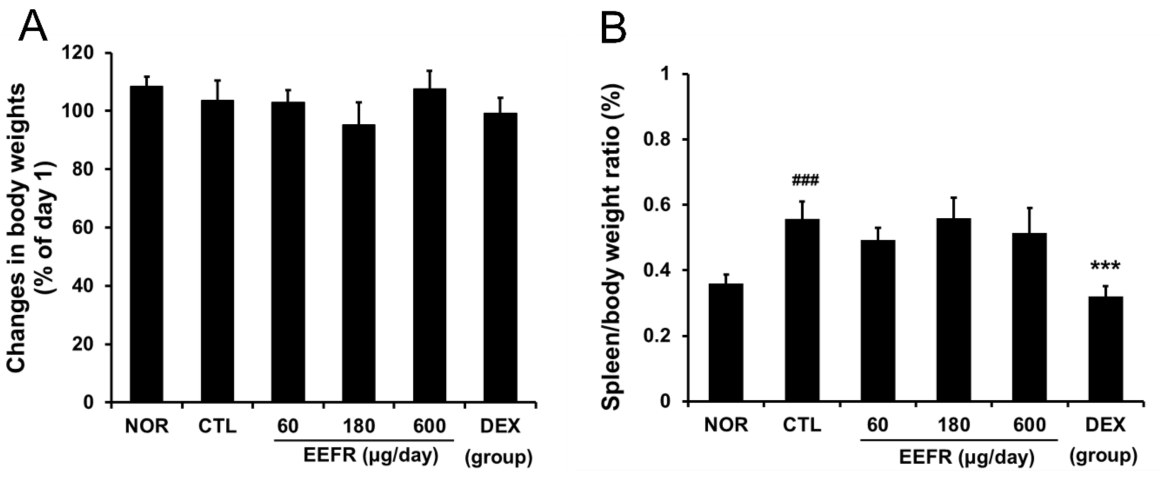

Figure S1. Effects of EEFR on spleen/body weight ratio in CD mice. Body and spleen weight were measured on day 15 and the spleen/body weight ratio was calculated. (A), changes in body weights; (B), spleen/body weight ratio. Abbreviations are same as Fig. 2. $^{###}P < 0.001$ vs. NOR; $^{***}P < 0.001$ vs. CTL.
