# Supplementary material for: The Related Mechanisms Predicted through Network-Based Pharmacological Analysis and the Anti-Inflammatory Effects of Fraxinus rhynchophylla Hance Bark on Contact Dermatitis in Mice
Source: Int J Mol Sci. 2023 Mar 23;24(7):6091. doi: 10.3390/ijms24076091 (PMC10094049; doi:10.3390/ijms24076091)
Supplement: Supplementary file 1 [file ijms-24-06091-s001.zip › ijms-2263352-supplementary/Supplementary Materials S5.pdf]

Supplementary data 1

Fraxinus rhynchophylla Hance (family Oleaceae) is a deciduous tree native to Mǎnzhōu (Northeast China) and Korean peninsula. The bark of *F. rhynchophylla* have been used to treat patients with inflammatory or purulent skin diseases in China, Japan and Korea. The bark of *F. rhynchophylla*, belongs to heat clearing herbs in the theory of traditional medicine, can clear away heat and has bitter taste and cold in nature. All photographs were taken at the Hwamyeong Arboretum located in Busan, South Korea.

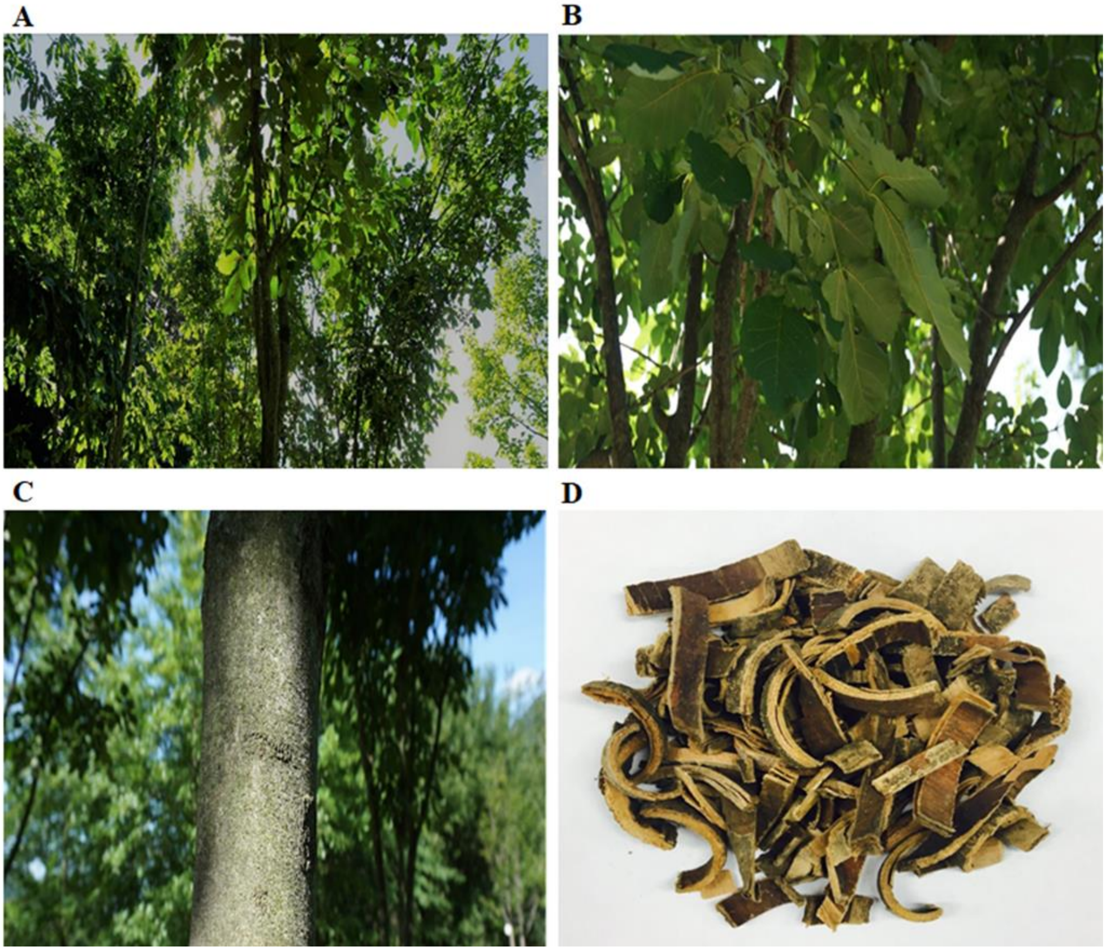

Figure S1. *Fraxinus rhynchophylla* Hance bark. Overall appearance (**A**), the leaves (**B**), bark (**C**) and dried bark of *Fraxinus rhynchophylla* (**D**).

Preparation of standard solution

Seventy percent ethanol extract of *F. rhynchophylla* bark (EEFR) was dissolved in methanol at 10 mg/ml. esculin and esculetin (standard compounds) were dissolved in methanol at each concentration of 0.5 mg/ml. EEFR and standard solution were filtered through 0.20 μm Syringe Filter (Bio FACT TM, Daejeon, Korea).

Chromatographic conditions

Agilent 1200 (Agilent Technologies, Palo Alto, CA, USA) HPLC equipped with an auto sampler, a column oven, a degasser, quaternary solvent pump and diode array detector (DAD) was used. The data were obtained by ChemStation (Agilent Technologies, Palo Alto, CA, USA). A 5 μl of sample and standard solution were injected into HPLC. The marker compounds in extracts were separated on a Zorbax eclipse XDB-C18 column (4.6 mm × 150 mm, 5 μm; Agilent, Palo Alto, CA, USA) at 35°C. The mobile phase consisted of acetonitrile and 0.1% acetic acid in water (table 1). The flow rate was set at 1.0 ml/min for 50min. UV wavelength was set at 340nm (Ahn et al., 2013).

Table. S1. Mobile phase used for the HPLC analysis

Retention time (min)	0.1% Acetic Acid in Water (%)	Acetonitrile (%)
0	94	6
20	90	10
25	80	20
30	80	20
35	70	30
40	94	6
50	94	6

Identification of esculin and esculetin in EEFR

The peaks of esculin and esculetin were detected in EEFR. The retention times of esculin and esculetin were 8.440 min and 13.513 min, respectively. In EEFR, esculin and esculetin peaks were also detected at the same retention time with standard solution (Fig. 2).

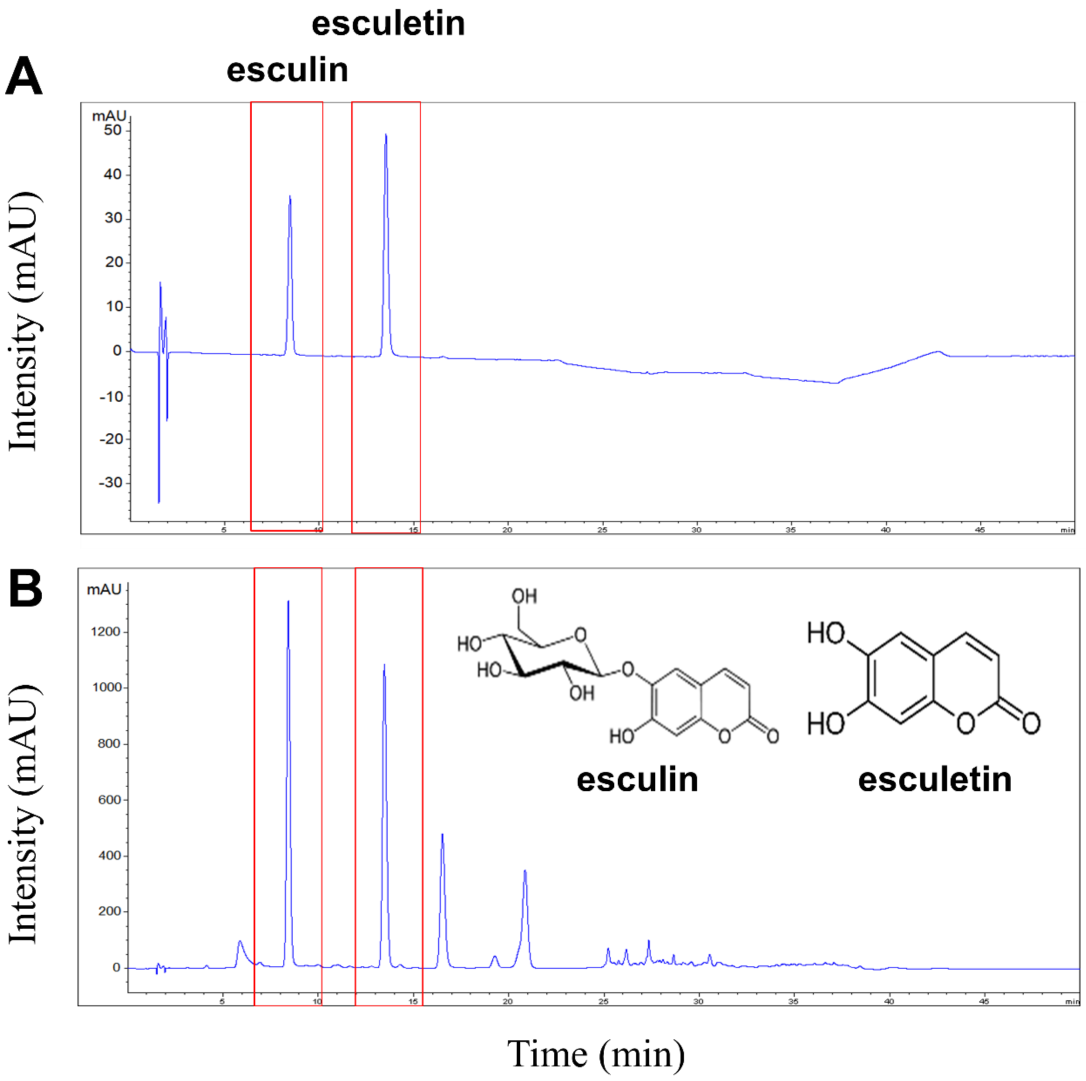

Figure S2. Chromatograms of esculin and esculetin Chromatograms of esculin and esculetin in standard solution (A) and EEFR (B) at UV wavelength of 340 nm.
