# Supplementary material for: The Related Mechanisms Predicted through Network-Based Pharmacological Analysis and the Anti-Inflammatory Effects of Fraxinus rhynchophylla Hance Bark on Contact Dermatitis in Mice
Source: Int J Mol Sci. 2023 Mar 23;24(7):6091. doi: 10.3390/ijms24076091 (PMC10094049; doi:10.3390/ijms24076091)
Supplement: Supplementary file 1 [file ijms-24-06091-s001.zip › ijms-2263352-supplementary/Supplementary Materials S6.pdf]

Supplementary data 2.

CD was induced by DNFB in Balb/c mice as previously described (Yang et al., 2019). Briefly, mice were sensitized with 30 μl of DNFB (0.2%, v/v) in acetone: olive oil (AOO, 4:1) onto both ears for three consecutive days. After four days of sensitization, the dorsum of all mice were shaved. On day 8, each mouse was challenged with 60 μl of DNFB (0.1% v/v) in AOO onto the shaved dorsum every two days (four times applications in total).

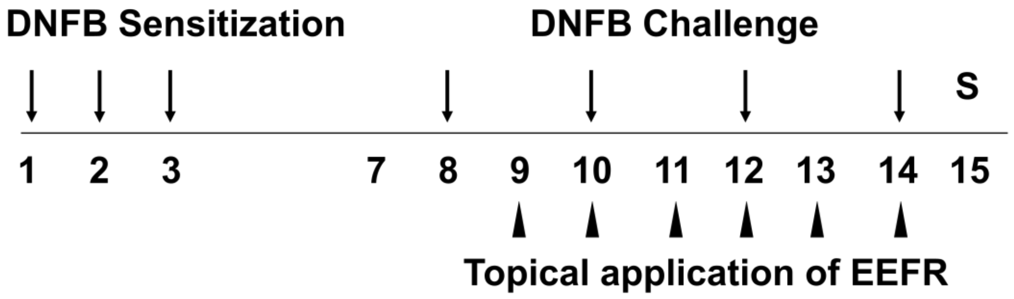

Figure S1. Experimental schedule. DNFB was applied onto the surface of each ear for sensitization on days 1, 2 and 3. Challenging was conducted using 0.1% of DNFB on days 8, 10, 12 and 14. EEFR and dexamethasone were topically applied for 6 consecutive days. All animals were sacrificed on day 15. EEFR, 70% ethanol extract of *F. rhynchophylla* bark; DNFB, 1-fluoro-2,4-dinitrobenzene; S, sacrifice.

Reference

Yang, B., Kim, S., Kim, J.H., Lim, C., Kim, H., Cho, S., 2019. Gentiana scabra Bunge roots alleviates skin lesions of contact dermatitis in mice. J. Ethnopharmacol. 233, 141-147.
