# Supplementary material for: The Related Mechanisms Predicted through Network-Based Pharmacological Analysis and the Anti-Inflammatory Effects of Fraxinus rhynchophylla Hance Bark on Contact Dermatitis in Mice
Source: Int J Mol Sci. 2023 Mar 23;24(7):6091. doi: 10.3390/ijms24076091 (PMC10094049; doi:10.3390/ijms24076091)
Supplement: Supplementary file 1 [file ijms-24-06091-s001.zip › ijms-2263352-supplementary/Supplementary Materials S7.pdf]

The following primer sets were used:

TNF-α:

F, 5'-CTCTCATCAGTTCTATGGCC-3'

R, 5'-TTGAAGAGAACCTGGGAGTA-3';

IL-1β:

F: 5'-CAGGCAGTATCACTCATTGT-3'

R: 5'-CCAGCAGGTTATCATCATCA-3'

IL-6:

F, 5'-GCCAGAGTCCTTCAGAGAGA-3'

R, 5'-GGTCTTGGTCCTTAGCCACT-3'

IL-8:

F, 5'-CATCCAGAGCTTGAGTGTGA-3'

R, 5'-GTTAGCCTTGCCTTTGTTCA-3'.

COX-2:

F, 5'-CATGGGTGTGAAGGGAAATA-3'

R, 5'-CGCAGTTTATGTTGTCTGTC-3'

GAPDH:

F, 5'-GATGACATCAAGAAGGTGGT-3'

R, 5'-TACCAGGAAATGAGCTTGAC-3'
